# Dissimilar Effects of Selenite and Selenium Nanoparticles on Skeletal Muscle Development Unrelated to GPx1 Activity During Adolescence in Rats

**DOI:** 10.3390/nu17111841

**Published:** 2025-05-28

**Authors:** Fátima Nogales, Eloísa Pajuelo, María del Carmen Gallego-López, Inés Romero-Herrera, Francisco Merchán, Olimpia Carreras, María Luisa Ojeda

**Affiliations:** 1 Departamento de Fisiología, Facultad de Farmacia, Universidad de Sevilla, 41012 Sevilla, Spain; fnogales@us.es (F.N.); mgallego3@us.es (M.d.C.G.-L.); iromero3@us.es (I.R.-H.); olimpia@us.es (O.C.); ojedamuri11@us.es (M.L.O.); 2 Departamento de Microbiología y Parasitología, Facultad de Farmacia, Universidad de Sevilla, 41012 Sevilla, Spain; fmerchan@us.es

**Keywords:** selenite, nanoparticles, skeletal muscle, glutathione peroxidase

## Abstract

**Background/Objectives**: During adolescence, the critical growth period, the antioxidant selenium (Se), either as sodium selenite or selenium nanoparticles (SeNPs), has shown contrasting effects on adipose tissue (AT) in rats, due to its role in insulin signaling. Since skeletal muscle (SKM) is also a key insulin-target tissue, this study aimed to assess whether a similar effect occurs in this tissue. **Methods**: Three groups of male adolescent rats (*n* = 18) were used: control (C), selenite supplemented (S), and SeNPs supplemented (NS). Low doses of Se were administered via drinking water in both supplemented groups. AT was utilized for transcriptomic analyses, while SKM was analyzed for oxidative balance, insulin-induced anabolic effects, and proteolysis. Myokine levels in serum were also determined. **Results**: SeNPs administration decreased SKM mass and protein content, increased serum creatinine, and decreased insulin levels, indicating impaired SKM development. Both supplemented groups upregulated genes related to creatine metabolism and muscle contraction. However, only the NS group showed upregulation of genes associated with glycogenolysis and glycolysis. Despite unchanged GPx1 expression, NS rats presented lower oxidation and insulin–pmTOR activation, and higher expression of proteins related to proteolysis (pAMPK, SIRT1, ULK1, FOXO3a, and MaFbx) and a myokine profile compatible to muscle atrophy, fatty acid oxidation, and impaired myoblast proliferation. Ultimately, the selenite group impaired SKM catabolism mainly by increasing insulin–pmTOR activation. **Conclusions**: Once again, the form of Se administration exerts opposing effects on metabolism tissues, suggests a potential therapeutic role for selenite in disorders that compromise muscle growth, such as muscular dystrophies, cachexia, or sarcopenia.

## 1. Introduction

Adolescence is a critical life period of growth and endocrine changes, marked by rapid physical, hormonal, and neurodevelopmental transformations that significantly influence body weight and composition [1]. These shifts are clearly manifested in the development of adipose tissue (AT) and skeletal muscle (SKM) mass. When this stage of growth and endocrine maturation is disrupted, obesity and insulin resistance (IR) could appear [2]; conversely, anorexia nervosa may also develop [3]. Lately, the prevalence of these disorders is dramatically increasing during adolescence [2,3]. Moreover, these pathologies share the common fact that insulin-target tissues—adipose tissue (AT), skeletal muscle (SKM), and liver—are affected, compromising health during this critical developmental period [4,5].

Selenium (Se) is a vital trace element known for its antioxidant and anti-inflammatory properties, which are largely mediated by selenoproteins like glutathione peroxidases (GPx) [6]. Beyond its antioxidant role, Se is increasingly recognized as a key regulator of the endocrine system, influencing hypothalamic–pituitary–peripheral feedback circuits, the thyroid hormone axis, glucoregulatory and adrenal hormones, the gonads in both sexes, the musculoskeletal system, and the skin [7]. The 25 identified selenoproteins intricately regulate the endocrine system and intracellular signaling, including GPx1, GPx3, GPx4, thioredoxin reductases (TXNRDs), Deiodinases (DIO 1–3), endoplasmic reticulum-resident SELS or SELW, and the hepatokine SELP [8]. Selenium regulates cell growth, metabolism and the endocrine function, since a proper oxidative balance is essential for the endocrine signaling and for cell proliferation/differentiation [9].

Our research group has focused on the study of Se supplementation on white adipose tissue (WAT) function, insulin secretion, and related molecular mechanisms in adolescent male rats [10,11,12]. Our approach consisted of employing two different forms of Se supplementation, soluble selenite or selenium nanoparticles (SeNPs), administered at the same concentration.

**Figure 1:**
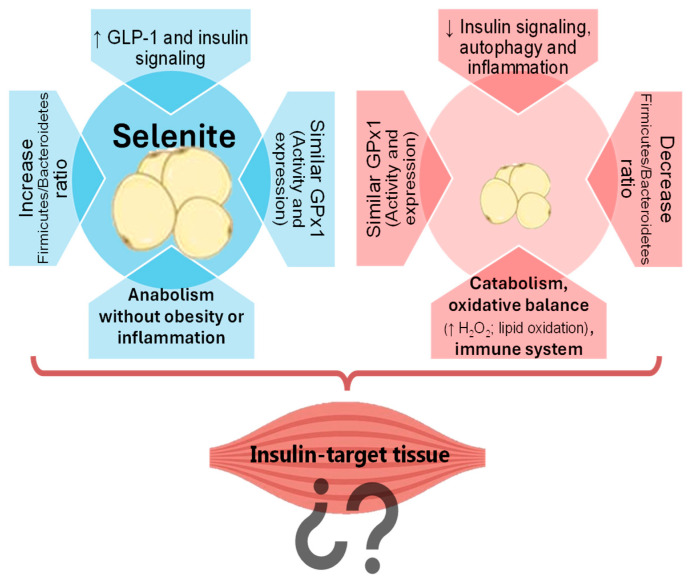
Contrasting effects of selenite and SeNPs on white adipose tissue: Exploring the implications for skeletal muscle.

Our studies have demonstrated the contrasting effects of selenite and Se NPs supplementation on AT. In fact, selenite supplementation was shown to promote adipogenesis via the insulin signaling pathway in WAT (Figure 1). Conversely, SeNP administration prevented fat accumulation in WAT by decreasing insulin signaling and promoting FOXO3a-mediated autophagy, a cellular recycling process, leading to reduced inflammation [10]. Notably, these effects were independent of GPx1 expression or activity. This selenoprotein has the lowest Se hierarchy, being highly sensitive to Se status; therefore, it is usually used to evaluate tissue Se levels [13]. The similar GPx1 values found in both groups confirm that both treatments used the same dose of Se, and that alternative mechanisms are involved [10]. In this regard, the possibility of an implication of the microbiota–liver–bile salt axis as a novel mechanism underlying the divergent effects of selenite and SeNPs on adipose tissue development was investigated. Selenite primarily affected the liver, decreasing the farnesoid X receptor (FXR) activity, and leading to bile salt accumulation, to an increased Firmicutes/Bacteroidetes ratio in the gut microbiota, and to a greater secretion of GLP-1. In contrast, SeNPs primarily impacted the gut microbiota, shifting it towards a more Gram-negative profile enriched in *Akkermansia* and *Muribaculaceae*, and decreasing the Firmicutes/Bacteroidetes ratio [11]. This microbial profile was associated with lower WAT mass, and SeNPs did not alter the pool of circulating bile salts. Transcriptomics of the WAT have shed some light on the mechanisms underlying the differential effects of selenite and SeNPs on gene expression in WAT [12]. SeNP supplementation led to a greater number of differentially expressed genes and impacted more cellular processes than selenite. Specifically, SeNPs upregulated genes associated with the immune system, catabolism, the mitochondrial function, and the oxidative balance. Gene ontology and KEGG pathway enrichment analyses revealed increased catabolic activity and decreased growth signaling in the SeNP group, contributing to a reduced WAT mass. Despite increased antioxidant enzyme activity, SeNP-treated rats also showed elevated H_2_O_2_ and malondialdehyde levels, indicating a complex interplay with oxidative stress (OS) [12]. These findings suggested a potential role for SeNPs in WAT reduction and immune response modulation during adolescence.

The transcriptomic analysis also revealed that, after Se treatments, a great number of genes related specifically to SKM function were differentially affected. SKM development is critical during adolescence [14] and is deeply related to the endocrine function and metabolism. Apart from its well-known contractile function and representing the 50% of the total human mass, this insulin-target tissue is the primary site for glucose disposal in response to insulin and the major reservoir of amino acids in the body [15]. Moreover, it is a secretory organ, synthesizing several myokines, which exert important paracrine, autocrine, and endocrine functions necessary for maintaining the general metabolic homeostasis [16,17]; interestingly, not all myokines are synthesized exclusively in this tissue. In addition, SKM contents nearly half of the total Se from the body, acting as a reservoir when there are deficiencies [18]. In the SKM, Se regulates various physiological functions; it has antioxidant, anti-inflammatory and anti-apoptotic properties [19]. Furthermore, Se has broader biological effects on SKM, being involved in energy balance and serving as a crucial regulator of myogenesis [18]. These roles highlight Se as a promising target for future therapies aimed at protecting and promoting SKM development.

Due to the fact that both WAT and SKM are tissues specially developed during adolescence and insulin-target tissues; and that transcriptomic analysis in WAT revealed that genes related to the SKM function were affected. In this work we will investigate whether the supplementation with either selenite or SeNPs can also have contrasting effects on the physiology of the SKM of adolescent rats, as happens in their WAT. Firstly, we will analyze specific genes related to the muscle tissue function, differently affected in WAT. Secondly, we will study different SKM parameters, such as mass, protein content, OS, the insulin signaling process, protein homeostasis, and myokine secretion.

## 2. Materials and Methods

### 2.1. Animals

In the present study, 18 adolescent male Wistar rats from Animal Production and Experimentation Centre of Vice-Rectorate for Scientific Research, of the University of Seville were used. At 21 days of age, they were brought in and kept in pairs with environmental enrichment for a one-week acclimation period. The experiment lasted three weeks, from postnatal day (PND) 28 to 47, corresponding to adolescence in Wistar rats [20]. The animals were kept in a climate-controlled room (22–23 °C) and 12:12 h light/dark cycle (light on 9:00 a.m.). All animal procedures were conducted in accordance with ethical standards and received prior approval from the Ethics Committee of the University of Seville (Approval Code: CEEA-US2019-4; Date: 27 February 2019) and the Andalusian Regional Authority (Approval Code: 05-04-2019-065; Date: 9 April 2019). The study complied with the guidelines set forth in European Union Directive 2010/63/EU and Spanish legislation as detailed in Royal Decree 53/2013 (BOE 34/11370).

At PND 28, experimental animals (*n* = 6/group; weight = 49.8 ± 3.3 g) were randomly distributed into the three following groups: control (C) (rats that received standard diet and water ad libitum); Selenite (S) (rats that received standard diet and selenite supplementation in water ad libitum); and SeNPs (NS) (standard diet and selenium nanoparticles supplementation in water ad libitum). This number of experimental units was carefully determined to minimize pain and distress, in accordance with the principles of replacement, reduction, and refinement.

Three groups had ad libitum access to a standard pellet diet (LASQCdiet^®^ Rod14-R, Märkische, Germani) which contained 0.2 ppm of Se provided as sodium selenite. The Se supplemented groups (S and NS) received an extra 0.14 ppm of Se through their drinking water throughout the experimental period. This supplementation was administered either as anhydrous sodium selenite (Panreac, Barcelona, Spain) or as SeNPs (developed at the Department of Organic and Medicinal Chemistry, Faculty of Pharmacy, University of Seville, Spain) [10].

### 2.2. Morphological and Biochemical Parameters, Samples

Throughout the experimental period, daily records were kept of the rats’ body weight as well as their intake of liquids and solids. Se consumption was estimated by multiplying the concentration of Se in food and water by the amount of food and water ingested daily. All measurements were taken at 9:00 a.m. to avoid changes due to circadian rhythm.

After a 12 h fasting period at the end of the experimental period, adolescent rats were weighed to record the final body weight and then subsequently anesthetized with an i.p. injection of 28% *w/v* urethane (0.5 mL/100 g body weight). Blood samples were collected through cardiac puncture and transferred into tubes for serum separation by centrifugation at 1300× *g* for 15 min. A midline abdominal incision was performed to obtain tissues. Retroperitoneal WAT and the gastrocnemius SKM from the right hindlimb were excised, weighed, frozen in liquid nitrogen and stored at −80 °C until later biochemical analyses were performed. The somatic index of SKM (SKMSI) was calculated as the ratio of muscle weight to body weight.

Creatinine and insulin levels were measured in the serum of adolescent rats using an automated analyzer (Technicon RA-1000, Bayer Diagnostics, Leverkusen, Germany).

The SKM homogenate was prepared in phosphate buffer (50 mM K_2_HPO_4_, 50 mM KH_2_PO_4_, 0.01 mM EDTA (Sigma-Aldrich, Madrid, Spain), pH 7.0) with a protease inhibitor at a 1:10 ratio (Complete Protease Inhibitor Cocktail Tablets, ROCHE, Madrid, Spain), using a Fisherbrand™ 850 homogenizer (Thermo Fisher Scientific Inc., Waltham, MA, USA) in an ice bath. Total protein levels in the SKM homogenate were determined using the Lowry method [21].

### 2.3. Transcriptomic Analysis

Total RNA was extracted from WAT samples from the corresponding experimental group (C, S, and NS) using the RNeasy Lipid Tissue Mini Kit (QIAGEN, Barcelona, Spain), following the manufacturer’s instructions [12]. RNA quality was analyzed by electrophoresis on a 1% agarose gel, and the presence of intact 18 S and 28 S ribosomal bands, along with a sufficient RNA quantity, was considered an indication of good RNA quality. All RNA samples were stored at −80 °C.

Messenger RNA was isolated from total RNA using oligo(dT)-coupled magnetic beads. Following fragmentation, first-strand cDNA synthesis was carried out using random hexamer primers, followed by second-strand synthesis employing dTTP, and generating a non-stranded cDNA library. The sequencing library was constructed through end repair, A-tailing, adapter ligation, size selection, PCR amplification, and purification, using a paired-end 150 bp protocol. Library quality control was performed using Qubit, real-time PCR, and a bioanalyzer prior to sequencing on the Illumina NovaSeq platform (Novogene, Cambridge, UK). Adapter sequences and low-quality reads were removed using the fastp software (v0.24.1) [22,23].

Reads were aligned to the *Rattus norvegicus* reference genome using HISAT2 (v2.0.5). The assembly was performed with StringTie (v1.3.3b), applying a genome-guide strategy. Read counts per gene were obtained using FeatureCounts (v1.5.0-p3), and expression values were reported in FPKM (Fragments Per Kilobase of transcript per Million mapped reads).

These expression levels were statistically analyzed using DESeq2 (v1.20.0), which identifies significantly and differentially expressed genes based on a negative binomial model. *p*-values were adjusted using the Benjamini–Hochberg method, considering genes with adjusted *p* ≤ 0.05 as differentially expressed. The analysis was conducted on two biological samples with 12 million high-quality paired end reads, showing high intra-sample correlation (mean: 93.8%). Gene expression changes were evaluated using the log2 fold change. Genes with |log2(FC)| ≥ 1.5 were considered significantly differentially expressed.

### 2.4. Oxidative Balance Analysis in SKM

To determine the oxidative balance in SKM, endogenous enzymes antioxidant activity, lipid and protein oxidation, levels of H_2_O_2_, and GPx1 and NOX4 expressions were assessed. The activity of superoxide dismutase (SOD), catalase (CAT), and glutathione peroxidase (GPx) was measured following the methods previously described [12,24]. Lipid oxidation in SKM was measured by quantifying malondialdehyde (MDA) levels (mol/mg protein) using the method of Draper and Hadley [25], and protein oxidation was assessed using the method of Reznick and Packer [26], which analyzes the carbonyl group levels in the sample (nmol/mg protein) through a reaction with 2,4-dinitrophenylhydrazine. H_2_O_2_ levels, as an indication of reactive oxygen species in this tissue, were measured using the Hydrogen Peroxide Colorimetric Detection Kit (Abnova, Taipei, Taiwan).

Finally, GPx1 and NOX4 proteins were analyzed by the Western blot technique according to the protocols used in our laboratory [24]. Specific primary antibodies were used at the following dilutions: GPx1:1:1000 (sc-133160; Santa Cruz Biotechnology, Santa Cruz, CA, USA); and NOX4:1:1000 (sc-518092; Santa Cruz Biotechnology, Santa Cruz, CA, USA). As secondary antibody Goat Anti-Mouse IgG (H + L) Horseradish Peroxidase Conjugate (catalog number 170-6516, BioRad, Hercules, CA, USA) was used in a 1:2500 dilution; as loading control, monoclonal mouse anti GAPDH (sc-32233, Santa Cruz Biotechnology, Santa Cruz, CA, USA) was used in a 1:1000 dilution.

### 2.5. Myokines

The serum levels of the myokines interleukin 6 (IL-6), myostatin, interleukin 15 (IL-15), fractalkine (CX3CL1), fibroblast growth factor 21 (FGF21), irisin, brain-derived neurotrophic factor (BDNF), follistatin-like protein 1 (FSTL-1) and fatty acid binding protein 3 (FABP-3), were quantified using the MILLIPLEX^®^ Rat Myokine Panel (Millipore Corp., St. Charles, MO, USA), utilizing xMAP technology (Luminex Corp., Austin, TX, USA) [27]. This multiplex immunoassay allows the simultaneous detection and quantification of multiple analytes in a single small-volume sample.

The samples, reagents, and standards were prepared according to Luminex xMAP protocol, using 25 μL of serum. Serum samples were incubated with magnetic beads pre-coated with analyte-specific capture antibodies. Following a series of washing steps to remove unbound material, biotinylated detection antibodies and streptavidin-phycoerythrin were added to generate a fluorescent signal. The intensity of the fluorescence was measured using a LABScan 100 analyzer (Luminex Corporation, Austin, TX, USA) with xPONENT software (v3.0) for data acquisition. Serum myokine levels were determined by comparing the mean fluorescence intensity of each duplicate sample against the corresponding assay’s standard calibration curve.

### 2.6. Statistical Analysis

The results related to morphology, nutrition, serum and SKM samples were presented as mean values with their corresponding standard error (±SEM). Statistical evaluations were performed using GraphPad Prism software (version 8.0.2; San Diego, CA, USA). Each experimental group consisted of six samples. A *p* value < 0.05 was considered statistically significant and assessed using one-way analysis of variance (ANOVA). When ANOVA indicated significant differences, post hoc analyses were conducted using the Tukey–Kramer test.

## 3. Results

Selenite or SeNPs treatments doubled Se intake (*p* < 0.001) in the S and NS rat groups, demonstrating that both supplementations were similar (Table 1). However, the muscle organosomatic index and total protein levels were significantly lower in SeNP-treated rats compared to the C group (*p* < 0.01 and *p* < 0.001, respectively) and the S group (*p* < 0.001 vs. C; *p* < 0.05 vs. CSe), although body weight gain was similar in all groups. These results suggested that SeNPs treatment provoked muscle degradation, as indicated by elevated creatinine levels in these rats compared with the rest of the groups (*p* < 0.001). In these SeNPs treated rats, it is also observed that there is a decrease in insulin levels compared to the C and S group (*p* < 0.05 and *p* < 0.01, respectively). By contrast, S group had higher insulin serum levels than C group (*p* < 0.05).

Table 2 shows the list of differently expressed genes in adipocytes related specifically or preferably to SKM. Some of these genes can be expressed also in the cardiac muscle, but not in the smooth tissue, so the expression level of these genes cannot be assigned to the smooth muscle of the blood vessels of the adipose tissue. Thus, when analyzing the gene expression profile in adolescent S rats, five genes were identified as significantly upregulated compared to C rats. These genes were involved in creatine metabolism (*Ckm*) and muscle contraction (*Acta1*, *Myh1*, *Myh7* and *Mylpf*) and presented changes in their expression levels ranging from 2.3- to 6.7-fold change (log_2_FC). The gene *Ckm* exhibited the highest upregulation.

Compared to the control, SeNPs treatment resulted in the significant upregulation of nine genes. These genes were involved not only in creatine metabolism (*Ckm*) and muscle contraction (*Acta1*, *Myh1*, *Myh7* and *Mylpf*) but also in the glycolysis pathway (*Pgam2*, *Eno3*), glycogen metabolism (*Pygm*), and Ca^2^+ transport (*Casq1*). All of them exhibited expression level changes ranging from 1.8 to 10.9 log_2_ fold change (log_2_FC). SeNPs treatment presented the highest log_2_FC values among the groups’ comparison. *Ckm* showed the highest upregulation.

However, the comparison between selenite and SeNPs treatments resulted in the downregulation of seven genes related to SKM function. In this comparison, genes involved in muscle contraction (*Acta1*, *Myh1*, *Myh7* and *Mylpf*) exhibited the greatest repression, very closely followed by those associated with creatine metabolism (*Ckm*), and then by those related to glycolysis (*Pgam2*) and calcium transport (*Casq1*).

**Figure 2:**
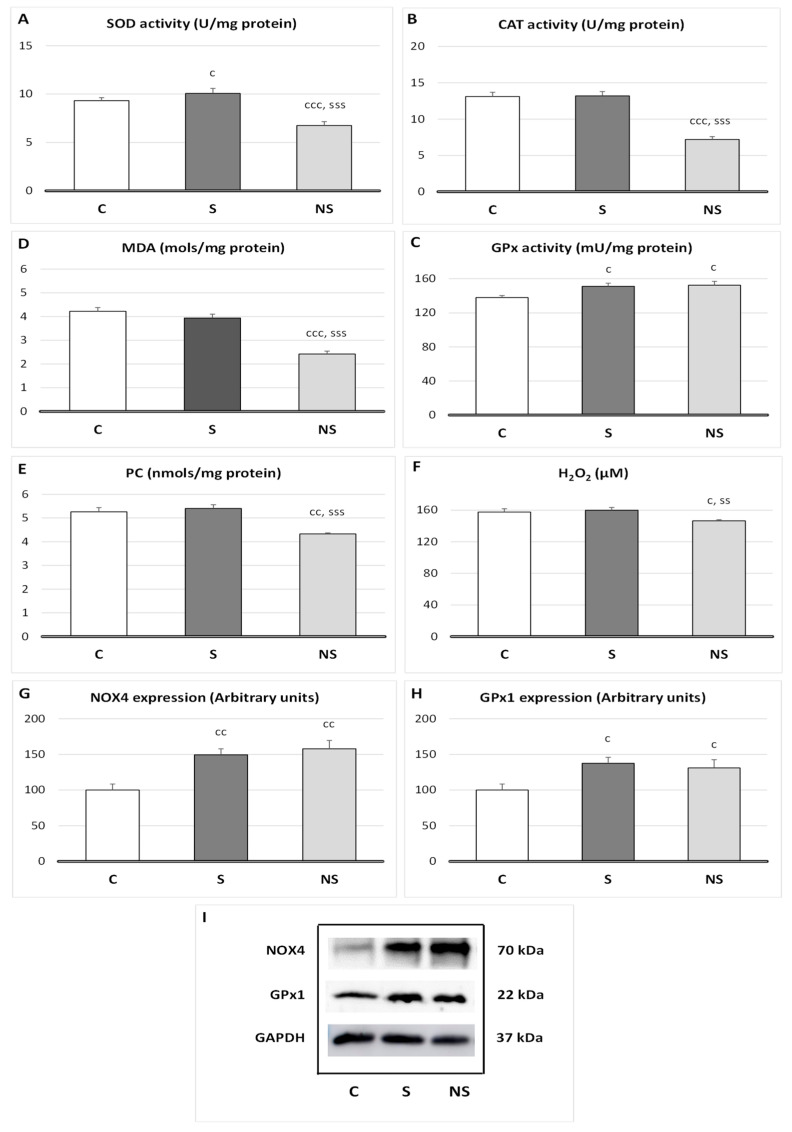
Oxidative balance in skeletal muscle of adolescent rats after treatment with selenite or SeNPs. (**A**) Superoxide dismutase (SOD) activity; (**B**) catalase (CAT) activity; (**C**) glutathione peroxidase (GPx) activity; (**D**) malondialdehyde levels (MDA), showing lipid peroxidation; (**E**) protein carbonyl group (PC), showing protein oxidation; (**F**) hydrogen peroxide (H_2_O_2_) concentration, a reactive oxygen species (ROS); (**G**) NADPH oxidase 4 (NOX4) expression; (**H**) glutathione peroxidase 1 expression; and (**I**) Western blot expression images with GAPDH as load control. The results were expressed as means ± SEMs and analyzed using a multifactorial one-way ANOVA followed by Tukey’s test. The number of animals used in each group is *n* = 6. Experimental groups: C, control group; S, selenite group; NS, SeNP group. Significance: vs. C, c *p* < 0.05, cc *p* < 0.01, ccc *p* < 0.001; vs. S, ss *p* < 0.01, sss *p* < 0.001.

Relative to oxidative balance in SKM (Figure 2), selenite treatment increases SOD (*p* < 0.05), GPx1 (*p* <0.05) activities, and GPx1 (*p* < 0.05) and NOX4 (*p* < 0.01) protein expressions, without affecting lipid or protein oxidation. SeNPs also showed an increase in GPx1 (*p* < 0.05) activity, GPx1 (*p* < 0.05), and NOX4 (*p* < 0.01) expressions. Nevertheless, SeNPs decreased SOD and CAT activities (*p* < 0.001 vs. C and S), MDA (*p* < 0.001 vs. C and S), PC (*p* < 0.01 vs. C, *p* < 0.001 vs. S), and H_2_O_2_ (*p* < 0.05 vs. C, *p* < 0.01 vs. S) levels in SKM.

**Figure 3:**
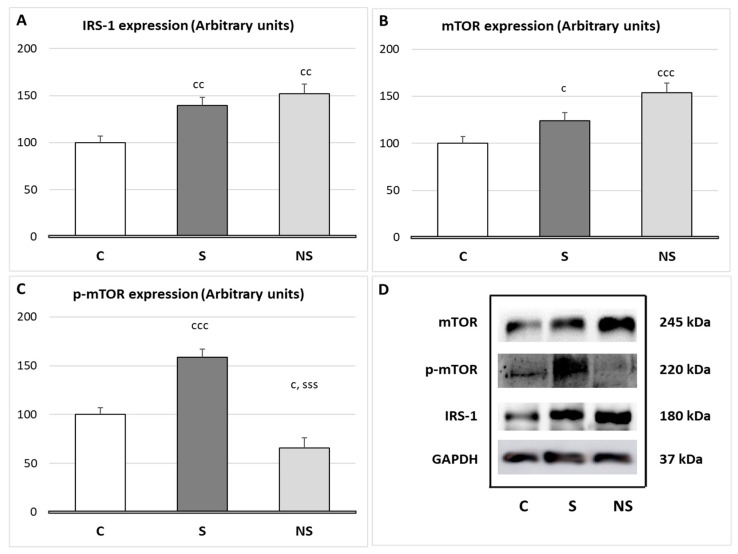
(**A**) Expression of the proteins Insulin receptor substrate 1 (IRS-1); (**B**) mammalian target of rapamycin (mTOR); (**C**) phospho-mTOR (Ser2448) (*p*-mTOR) after treatment with selenite or SeNPs in SKM; and (**D**) Western blot expression images with GAPDH as a loading control. The results were expressed as means ± SEMs and analyzed using a multifactorial one-way ANOVA followed by Tukey’s test. The number of animals used in each group is *n* = 6. Experimental groups: C, control group; S, selenite group; NS, SeNP group. Significance: vs. C, c *p* < 0.05, cc *p* < 0.01, ccc *p* < 0.001; vs. S, sss *p* < 0.001.

Both experimental groups, S and NS, increased IRS-1 expression in SKM (*p* < 0.01). However, selenite significantly increased *p*-mTOR expression (*p* < 0.001 vs. C and NS), and SeNPs treatment increased mTOR expression (*p* < 0.001 vs. C, *p* < 0.05 vs. S) and decreased *p*-MTOR expression (*p* < 0.05 vs. C) (Figure 3).

**Figure 4:**
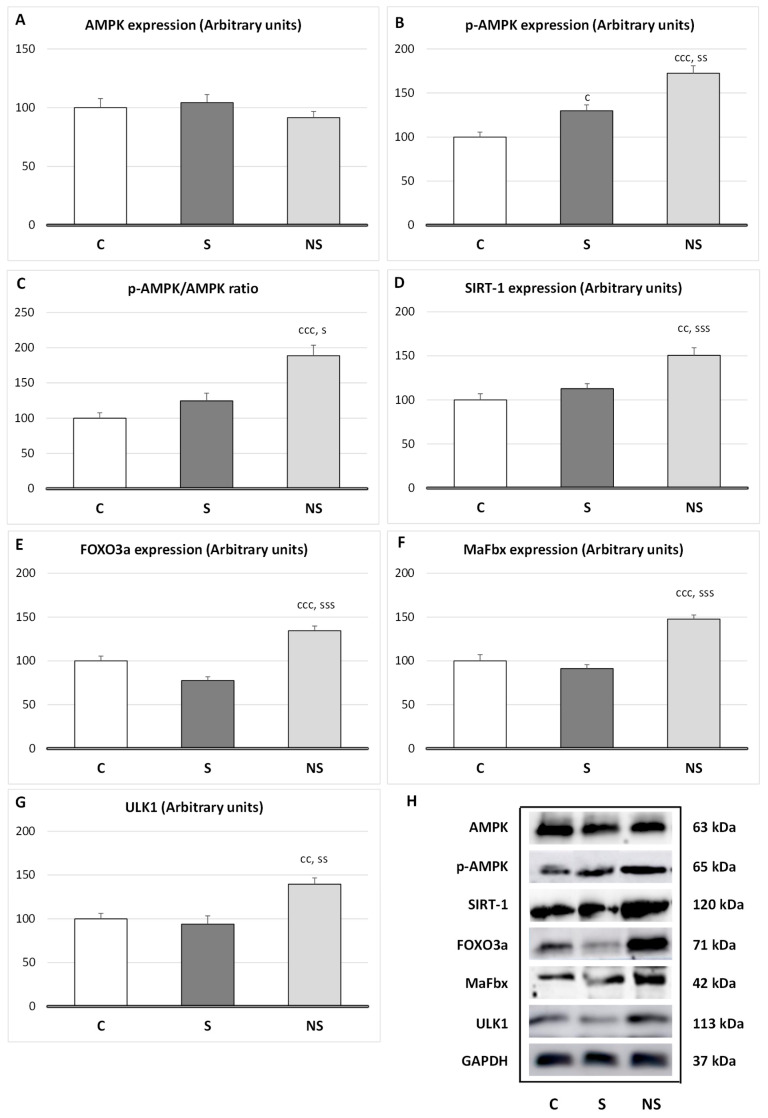
(**A**) Expression of total AMPK; (**B**) *p*-AMPK; (**C**) the *p*-AMPK/AMPK ratio; (**D**) additional expressions of SIRT-1; (**E**) FOXO3a; (**F**) MaFbx; and (**G**) ULK following treatment with selenite or SeNPs; and (**H**) Western blot images show protein expression with GAPDH as a loading control. The results were expressed as means ± SEMs and analyzed using a multifactorial one-way ANOVA followed by Tukey’s test. The number of animals used in each group is *n* = 6. Experimental groups: C, control group; S, selenite group; NS, SeNP group. Significance: vs. C, c *p* < 0.05, cc *p* < 0.01, ccc *p* < 0.001; vs. S, s *p* < 0.05, ss *p* < 0.01, sss *p* < 0.001.

Regarding protein degradation in SKM (Figure 4), selenite only increased the expression of *p*-AMPK (*p* < 0.05 vs. C); however, the ratio *p*-AMPK/AMPK was unaltered. By contrast, SeNPs enhanced the expression of *p*-AMPK (*p* < 0.001 vs. C, *p* < 0.01 vs. S) and its ratio (*p* < 0.001 vs. C), and the expression of SIRT-1 (*p* < 0.01 vs. C, *p* < 0.001 vs. S), FOXO3a (*p* < 0.001 vs. C and S), MaFbx (*p* < 0.001 vs. C and S) and ULK1 (*p* < 0.01 vs. C and S).

Relative to myokines secretion (Table 3), both treatments SNPs and selenite led to an increase in Il-6 (*p* < 0.05), and a decrease in LIF (S: *p* < 0.001; NS: *p* < 0.01), Apelin (*p* < 0.001) and SPARC (*p* < 0.01) serum levels vs. control rats. S adolescent animals showed an enhancement in FGF21 (*p* < 0.001), FSTL-1 (*p* < 0.01) and FABP3 (*p* < 0.01 vs. C; *p* < 0.001 vs. NS) levels. NS rats presented higher levels of serum IL-15 vs. C ones (*p* < 0.05), and BDNF vs. C (*p* < 0.01) and vs. S (*p* < 0.05) animals.

## 4. Discussion

### 4.1. Se Supplementation Modulates Genes Related to Muscle Function in Adipocytes by Upregulating Creatinine Kinase and Muscle Contraction Genes—These Effects Are Higher in SeNPs Treated Rats

Previously, we observed that in WAT either selenite or SeNPs supplementation had different effects on transcriptomic studies, mainly by SeNP’s upregulation of genes implicated in the immune system, catabolism, the mitochondrial function, the oxidative balance, and even in muscle tissue function. Despite the fact that adipocytes are not contractile cells, they can express genes related to SKM contraction. This can happen because mesenchymal cells, from which adipocytes, myocytes, osteocytes, and chondrocytes are derived, have high cellular plasticity and the capacity to differentiate into different types of cells depending on external factors, such as hormones [28,29]. The adipocyte gene expression related to SKM contraction, such as actin or myosin, is a clear example of how adipocytes can adapt and response to external signals [30].

In this study, we have found that Se supplementation, regardless of its form of administration (either selenite or SeNPs), enhanced the expression of the *Ckm* gene, which encodes creatine phosphokinase (CPK), the predominant in skeletal muscle (SKM) in adipocytes. This enzyme is essential for rapidly regenerating ATP, by transferring a phosphate group from phosphocreatine (PCr) to ADP, being the energy source the PCr. It is extremely important for explosive, high-intensity activities since ATP is generated in 10–15 s [31]. These data indirectly indicate that Se provides a rapid energy supply in SKM, which is greater when administered as SeNPs. Se supplementation in both forms also upregulates the genes which encode the proteins related to SKM contraction: alpha-actin of SKM, a key structural protein in muscle contraction (gen *Acta1*); myosin heavy chain 1 of SKM, one of the myosin isoforms responsible for muscle contraction in fast fibers (gen *Myh1*); myosin heavy chain 7 of SKM, one of the myosin isoforms responsible for muscle contraction in slow fibers (gen *Myh7*); and myosin light chain specific to fast SKM, which regulates myosin activity in muscle contraction (gen *Mylpf*) [32,33]. In all cases, the upregulation of these genes was significantly greater after SeNPs-treatment. These results indicate that Se enhances SKM contraction process, especially in the form of SeNPs.

Moreover, SeNPs also enhanced specific genes from SKM related to glycolysis (*Pgam* and *Eno3*), to glycogen degradation (*Pygm*), and to calcium transport (*Casq1*). These genes are involved in anaerobic glycolysis, which converts glucose into pyruvate, providing ATP quickly, but leading to muscle fatigue due to lactate accumulation if oxygen is insufficient. This process generates ATP in 30 s to 2 min [34]. In this context, *Pgam* encodes the enzyme phosphoglycerate mutase (PGAM), which catalyzes the conversion of 3-phosphoglycerate to 2-phosphoglycerate, and *Eno3* encodes beta-enolase (ENO3), another glycolytic enzyme that converts 2-phosphoglycerate to phosphoenolpyruvate, contributing to ATP generation in SKM for muscle contraction. Finally, *Pygm* encodes glycogen phosphorylase, an enzyme that breaks down glycogen into glucose-1-phosphate, providing fuel for glycolysis in SKM, and therefore for PGAM and ENO3. These proteins together ensure a higher supply of ATP for SKM contraction in SeNPs treated animals. Moreover, *Casq1* encodes calsequestrin-1, a calcium-binding protein in the sarcoplasmic reticulum, which regulates calcium release for muscle contraction, contributing to a proper calcium excitation-contraction signaling. Together, these genes indicate that during SeNPs treatment, energy is supplied by generating ATP via PCr system and through anaerobic glycolysis. It is known that fast fibers have lower amount of mitochondria and usually activate PCr system and anaerobic glycolysis to obtain ATP [35]. In this study, the upregulation of *Myh1* and *Mylpf,* which encode for proteins responsible for muscle contraction in fast fibers, is in consonance with these results, indicating that SeNPs promote fast muscle fibers contraction.

Finally, the most efficient pathway generating ATP per glucose molecule in the SKM is the oxidative phosphorylation. It occurs in the mitochondria, uses oxygen, and is crucial for endurance activities, lasting its effects from minutes to hours. It is the main energy source of slow fibers, which also have a greater number of mitochondria than fast fibers [36]. Since SeNPs treatment increases genes related to slow fibers contraction (*Myh7*) in adipocytes approximately eightfold more than in C rats, this energy pathway should also be enhanced in these animals.

During oxidative phosphorylation, reactive oxygen species (ROS) are produced naturally as a byproduct of the electron transport chain. Although under physiological conditions this generation is discrete, under stress conditions, when mitochondrial activity is increased, these levels can reach excessive values, leading to OS and to cell damage. For this reason, the endogenous antioxidant systems, superoxide dismutase (SOD), catalase (CAT) and glutathione peroxidase (GPx), which neutralize ROS to minimize oxidative damage, should be balanced. Therefore, we will analyze the oxidative balance in SKM.

### 4.2. SeNPs Decreases H_2_O_2_ Generation in SKM, Coinciding with Lower SKM Mass

The main known function of Se is its antioxidant activity, since it is part of the catalytic center of the antioxidant enzyme GPx. Along with CAT and SOD, GPx is one of the three main antioxidant endogenous enzyme in the body to combat ROS, such as the superoxide anion and hydrogen peroxide (H_2_O_2_); consequently, they are essential for maintaining a correct oxidative balance. In SKM, oxidative balance plays a pivotal role in myogenic proliferation and differentiation. For proper proliferation from satellite cells to myoblasts, low H_2_O_2_ concentrations are necessary. However, for differentiation from myoblasts to myotube formation, certain levels of H_2_O_2_ are required [37]. Moreover, it has been described that specifically an upregulation of GPx activity during proliferation, combined with appropriate H_2_O_2_ levels, enhances myotube formation [38].

In terms of oxidative balance, selenite supplementation to adolescent rats slightly increased SOD activity as well as GPx1 activity and expression, without affecting H_2_O_2_ concentration or lipid or protein oxidation in SKM. This final effect occurred because GPx1 specifically converts the H_2_O_2_ generated by SOD into H_2_O and O_2_. Moreover, to prevent a significant decrease in H_2_O_2_ concentration, which is necessary for differentiation and growth, the pro-oxidant enzyme NOX4 is upregulated, generating physiological levels of H_2_O_2_. In fact, a physiological adaptive relationship among NOX4, GPx1 and H_2_O_2_ stability in SKM has been described [39]. For this reason, in selenite supplemented rats, SKM mass is physiologically developed. In previous papers, we have observed similar results in WAT mass of these animals [10].

Nevertheless, SeNPs supplementation, at the same dose as selenite, reduced the antioxidant activities of SOD and CAT. This decline in antioxidant activity was not associated with a higher oxidative profile, as lipid and protein oxidation remained below control values in SKM, indicating a decrease in ROS levels. Moreover, although GPx1 and NOX4 were enhanced, functioning together to maintain proper H_2_O_2_ levels, H_2_O_2_ SKM deposits were significantly reduced. This suggests the presence of an independent mechanism beyond GPx1 activity, which leads to this deep antioxidant effect. This mechanism should be likely related to lower ROS formation; for this reason, CAT and SOD activities were decreased.

Considering that mitochondria are highly abundant in SKM, especially in slow fibers, due to the high energy demand of muscle contraction [36], and that they serve as the main source of ROS in SKM [40], the transcriptional studies undertaken could provide further insights into this phenomenon. It is known that CPK, which is strongly upregulated in adipocytes treated with SeNPs, plays a crucial role not only in energy function but also in preventing mitochondria ROS generation through an ADP-recycling mechanism [41]. This mechanism could partially explain the strong antioxidant effect of SeNPs in SKM and the lower H_2_O_2_ levels detected in SeNPs-treated rats, which may impair a correct differentiation process, coinciding with a reduced SKM mass.

### 4.3. SeNPs Deeply Affect Proteosynthesis and Proteolysis Leading to Protein Loss

The regulation of SKM mass is mainly controlled by a fine balance between protein synthesis (proteosynthesis) and protein degradation (proteolysis). In this context, after selenite supplementation, the expression of IRS-1 in SKM was upregulated, similar to what we observed previously in WAT [10], leading to the activation of the IRS-1/Akt/mTOR pathway, the main regulator of proteosynthesis [42]. Since these rats also presented higher insulin serum levels, the activation of this pathway was ensured. It is well described that selenite acts as a secretagogue of insulin through various mechanisms [43], such as increasing the incretin GLP-1 [11]. However, these animals presented normal protein content in SKM, since, as it will be analyzed later, their myokine profile involves catabolic effects. Regarding proteolysis, despite the activation of IRS-1/Akt/mTOR pathway, which inhibits proteolytic processes by suppressing FOXO3 (responsible for autophagy and the ubiquitin–proteasome system) [44], selenite did not reduce any of the proteolytic routes studied, as these remained unaffected.

By contrast, despite SeNPs miocytes presenting higher IRS-1 expression than those treated with selenite, they did not activate the IRS-1/Akt/mTOR pathway. This could be due to two main reasons. First, these animals have lower serum insulin levels compared to physiological conditions. Second, H_2_O_2_ acts as a second messenger in the insulin pathway [39], and in this group of animals its levels are decreased. Since SeNPs reduced H_2_O_2_ levels in SKM, insulin sensitivity is compromised. These data are in consonance with the high activity found in fast fibers, since these fibers are less dependent on insulin and glucose uptake from blood, unlike slow fibers [45]. Fast fibers rely on PCr system and anaerobic glycolysis; in this context, in insulin-resistant individuals, a higher recruitment of fast fibers has been described [46].

Moreover, like in WAT, catabolic pathways related to proteolysis are increased following SeNPs exposure [10,12]. Both crucial cellular energy sensors, AMP-activated protein kinase (AMPK) and NAD^+^-dependent deacetylase sirtuin-1 (SIRT1), which are activated when energy is necessary, were enhanced [47]. In fact, these sensors activate different catabolic routes to obtain energy, leading to proteolysis. These data align with the lower protein content and reduced SKM mass found in the SeNPs-treated group.

AMPK enhances its activity when ATP levels are required, and in order to obtain energy initiates different routes to avoid anabolism, for example, by inhibiting IRS-1/Akt/mTOR pathway [48], and activates catabolic routes such as ubiquitin–proteasome (UPS) route via MaFbx/atrogin-1 and the autophagic-lysosomal system by activating unc-51-like kinase 1(ULK1), increasing proteolysis [49].

Moreover, SeNPs enhanced SIRT1 expression, this energy sensor is also activated when energy is required and is intrinsically linked to cellular metabolism [50]. SIRT1 usually acts in coordination with AMPK, but it also exhibits its own properties. For instance, in SKM, SIRT1 promotes myogenic proliferation, while preventing differentiation by decreasing MyoD expression [51]. Additionally, it downregulates insulin signaling and enhances fatty acid oxidation and mitochondrial biogenesis by inducing PGC-1alfa and FOXO3a [52]. In this context, by activating FOXO3a, SIRT1 induces MaFbx expression and proteolysis via the UPS, and the autophagic–lysosomal system [53]. Finally, SIRT1, mainly by deacetylating various proteins, contributes to cellular antioxidant responses. For example, it deacetylates FOXO3 and PGC-1alfa, which contribute to induce the activity of antioxidant enzymes, and to the reduction in ROS generation [54,55]. The enhancement of SIRT1 and FOXO3a could be another point to take into consideration in the high antioxidant capacity observed after SeNPs-treatment in SKM.

It is evident that SeNPs, by decreasing H_2_O_2_ levels, affect myogenic differentiation and IRS-1/Akt/mTOR activation, thereby avoiding proper protein synthesis and SKM mass development. Moreover, SeNPs increase catabolic process in SKM through different pathways (AMPK, SIRT1 and FOXO3a), which increase MaFbx and ULK1 expression, and therefore the UPS and autophagy system. These results align with the highest creatinine serum levels found in these animals.

Finally, SKM is a highly ductile organ, which has the capability to adapt to various physiological conditions by changing its size, composition, and metabolic properties. Therefore, to determine whether these catabolic changes have repercussions on the serum myokine profile, the main myokines were analyzed.

### 4.4. Different Myokine Secretion Pattern After Selenite or SeNPs Administration

**Figure 5:**
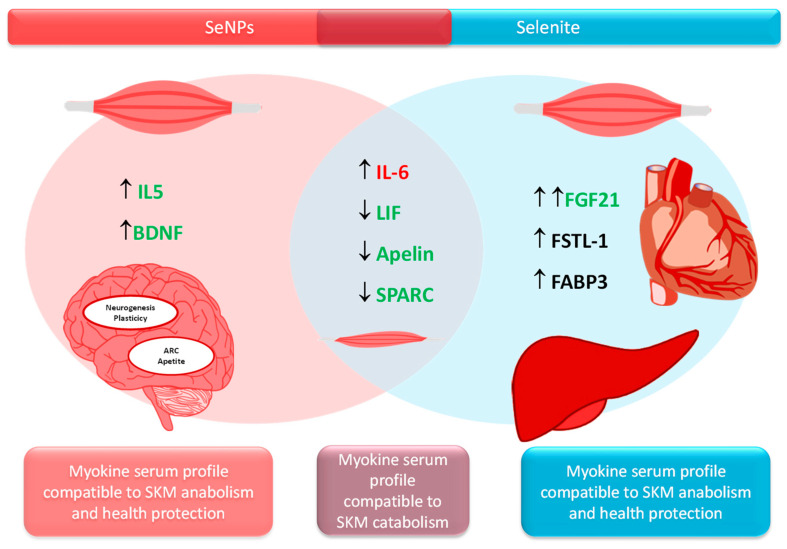
The Venn diagram shows the contrasting effects of Selenite and SeNPs supplementation on the levels of myokines. Whereas, some myokines such as IL-6, LIF, Apelin and SPARC showed similar behavior in both treatments, others such as FGF21, FSTL-1 and FABP3 are much induced in NS rats, and IL-15 together with BDNF are induced specifically in S rats. Other target organs for these contrasting myokines are indicated. An ↑ indicates an increase, an ↓ indicates a decrease. A double mark indicates a more significant change.

Both treatments selenite and SeNPs alter the homeostasis of several myokines towards a catabolic profile (increased Il-6 and decreased LIF, Apelin and SPARC) which collaborate, among others, to muscle atrophy and fatty acid oxidation, impaired myoblast proliferation and compromised SKM repair and remodeling [56]. However, both treatments presented a different alternative mechanism to avoid muscle breakdown, being the strategy of the S group more potent, as shown in the results relative to SKM protein content, SKM mass and *p*-mTOR results (Figure 5).

It is important to underline that some myokines, as shown in Table 3, are also secreted by other tissues such as the liver and AT (insulin-target tissues), indicating that the endocrine crosstalk among the liver, SKM and AT is greater than expected [57,58]. Therefore, selenite significantly enhanced FGF21 serum levels, which is related to higher muscle mass and mitochondria biogenesis by increasing Akt/mTOR pathway [56]. In fact, it could be established that selenite increases muscle protein synthesis, among others, by promoting FGF21 synthesis. This myokine also has important endocrine actions, such as enhancing insulin sensitivity [16], protecting against cardiac hypertrophy [59], and it also has important beneficial roles in the liver, reducing fat accumulation, inflammation and fibrosis [60]. Selenite supplementation to adolescent rats also increased serum levels of myokines with important endocrine functions, such as FSTL-1 and FABP-3. The first one contributes to increasing glucose uptake and to enhancing β-cell function by improving insulin sensitivity. This is the case in our S rats, where IRS-1 and *p*-mTOR expressions are increased. FSTL-1 also attenuates liver fibrosis, increases endothelial function and revascularization, and improves cardioprotection [16]. FABP-3 acts as a fatty acid carrier specialized in supplying energy to the heart, critical for maintaining the homeostatic function of skeletal and cardiac muscles [61]. In fact, selenite supplementation has been found to be a useful strategy to avoid SKM and cardiovascular damage [62].

SeNPs treatment developed another tactic to increase muscle mass; however, in terms of SKM mass development, this was less efficient. This treatment increases IL-15 and BNDF serum levels. IL-15 contributes to myoblast differentiation, increases fat metabolism preventing fat depots, and enhances antioxidant capacity in SKM [16]. It also has important endocrine functions, and decreases visceral fat by increasing fat metabolism, but also by enhancing energy expenditure and mitochondrial function [63], promoting lean body weight regulation [64]. These effects of SeNPs in adolescent rats are consistent with those found in previous studies, where SeNPs treatment influenced the gut microbiota, shifting it towards a more Gram-negative profile enriched in *Akkermansia* and *Muribaculaceae*, both important bacterial families linked to weight loss, while also decreasing the Firmicutes/Bacteroidetes ratio, associated with a leaner body composition [11].

SeNPs also increased serum BNDF levels, which contribute to muscle regeneration and, as IL-15, to fatty acid oxidation in SKM. This catabolic effect of SeNPs is in consonance with the catabolic status found in the SKM of the rats used in this work. BDNF plays a significant role in memory and plasticity by boosting hippocampal neurogenesis, clearly indicating that SeNPs improve neural function. Finally, BDNF acts in the arcuate nucleus and decreases appetite [64].

These results demonstrate that both Se supplementations modulate SKM and AT morphology, metabolism, and function, clearly highlighting their crosstalk, which may affect energy metabolism and muscle contractility.

## 5. Conclusions

This study demonstrates that Se supplementation, either in form of selenite or SeNPs, differentially affects the expression of several specific genes, related to SMK, in adipocytes, suggesting a stimulation of fibers. However, SeNPs present a stronger effect in ATP generation by PCr system and anaerobic glycolysis, mainly improving the function of fast fibers; nevertheless, slow fibers also seem to be stimulated. Direct studies on SKM reveal that selenite and SeNPs have different effects on SKM, reducing SeNPs SKM mass. This detriment is mainly due to the fact that SeNPs have a potent antioxidant activity, which decreases H_2_O_2_ SKM levels, potentially impairing muscle differentiation and an appropriate function of the IRS-1/Akt/mTOR pathway. These factors clearly hinder the proper growth and development of SKM mass. Moreover, SeNPs also activate catabolic routes through AMPK and SIRT1 upregulation, which are in consonance with the transcriptomic results related to higher muscle contraction founded. Finally, SeNPs alters the homeostasis of several myokines towards a catabolic profile, but increases serum IL-15 and BNDF levels, which contribute to muscle regeneration, and to fatty acid oxidation and neuronal plasticity, respectively. These effects of SeNPs on SKM are parallel to those in WAT mass. However, despite selenite treatment also altering some of the routes evaluated, its final effect was physiological, not affecting SKM mass. Further studies are needed to confirm these findings in humans and to clarify the long-term effects of Se supplementation. Selenite may be useful therapeutically in muscle-wasting conditions, such as muscular dystrophies or cachexia, while SeNPs, due to their stimulation of fast fibers and ATP production, could be relevant for enhancing muscle performance.

Limitations of the study. One of the main limitations of the present study is that the expression data for skeletal muscle-related genes were derived from transcriptomic analysis of WAT, rather than directly from SKM. Although adipocytes are not contractile cells, they can express genes related to SKM contraction. This can happen because mesenchymal stem cells, from which adipocytes, myocytes, osteocytes, and chondrocytes are derived, have high cellular plasticity and the ability to differentiate into various cell types in response to external factors, such as hormones. Moreover, it should be emphasized that all genes selected in this study are predominantly or exclusively expressed in SKM. Despite the fact that some of these genes can also be expressed in the cardiac muscle, none are expressed in smooth muscle; therefore, their expression in WAT cannot be attributed to the smooth muscle cells of blood vessels from WAT. The detection of these genes in WAT may instead reflect inter-organ communication mechanisms. Finally, to confirm and elucidate these findings, a comprehensive transcriptomic analysis using SKM is planned as the next step in this research.

## Figures and Tables

**Table 1:** Nutritional and Morphological Parameters in Adolescent Rats Following Treatment with Selenite or SeNPs.

	C	S	NS
Increased body weight (g/d)	6.01 ± 0.1	6.07 ± 0.2	5.98 ± 0.2
Total Se intake (µg/d)	3.48 ± 0.08	6.81 ± 0.14 ccc	6.59 ± 0.09 ccc
SKMSI (%)	0.67 ± 0.016	0.70 ± 0.022	0.58 ± 0.013 cc, sss
SKM protein (mg/g WT)	67.1 ± 2	63.7 ± 1.5	56.5 ± 1.3 ccc, s
Creatinine (mg/dL)	0.45 ± 0.005	0.43 ± 0.006	0.49 ± 0.006 ccc, sss
Insulin (mU/L)	0.019 ± 0.001	0.028 ± 0.004 c	0.013 ± 0.001 c, ss

The results were expressed as means ± SEMs and analyzed using a multifactorial one-way ANOVA followed by Tukey’s test. The number of animals used in each group is *n* = 6. Experimental groups: C, control group; S, selenite group; NS, SeNP group. Significance: vs. C; c *p* < 0.05; cc *p* < 0.01; ccc *p* < 0.001; vs. S; s *p* < 0.05; ss *p* < 0.01; sss *p* < 0.001. SKMSI: Skeletal muscle somatic index.

**Table 2:** List of statistically significant differentially regulated genes (Log_2_ fold change) related specifically to SKM after Selenite and SeNPs treatments in adipocytes.

Status	Gene_Symbol	Related Function	Log_2_FC
**UPREGULATED** **by Selenite vs. Control**	*Ckm*	Creatine metabolism	6.7
	*Acta1*	Muscle contraction	4.9
	*Myh1*	Muscle contraction fast fibers	4.8
	*Myh7*	Muscle contraction slow fibers	3.7
	*Mylpf*	Muscle contraction fast fibers	2.3
**UPREGULATED** **by SeNPs vs. Control**	*Ckm*	Creatine metabolism	10.9
	*Acta1*	Muscle contraction	9.1
	*Myh1*	Muscle contraction fast fibers	9.7
	*Myh7*	Muscle contraction slow fibers	8.5
	*Mylpf*	Muscle contraction fast fibers	6.9
	*Pgam2*	Glycolysis	2.7
	*Casq1*	Ca^2+^ channel transport	2.5
	*Eno3*	Glycolysis	1.9
	*Pygm*	Glycogen metabolism	1.8
**DOWN REGULATED by** **Selenite vs. SeNPs**	*Myh1*	Muscle contraction fast fibers	−4.8
	*Myh7*	Muscle contraction slow fibers	−4.8
	*Mylpf*	Muscle contraction fast fibers	−4.7
	*Acta1*	Muscle contraction	−4.1
	*Ckm*	Creatine metabolism	−4.2
	*Pgam2*	Glycolysis	−2.6
	*Casq1*	Ca^2+^ channel transport	−1.6

**Table 3:** Serum myokines levels in adolescent rats after treatment with selenite or SeNPs.

Myokine (pg/mL)	C	S	NP	Secretory Tissue
IL-6	0.31 ± 0.006	0.42 ± 0.03 c	0.42 ± 0.01 c	Muscle, AT, Liver, and Immune system
MYOSTATIN	393.2 ± 2.9	390 ± 4.4	401 ± 3.6	Muscle
LIF	7.2 ± 0.3	4.5 ± 0.4 ccc	5.4 ± 0.4 cc	Muscle
IL-15	1.09 ± 0.04	1.31 ± 0.06	1.39 ± 0.08 c	Muscle
FGF21	3.5 ± 0.15	14.85 ± 0.7 ccc	4.03 ± 0.1 sss	Muscle, AT and Liver
IRISIN	205 ± 5	199 ± 6	202 ± 1	Muscle
BDNF	3527 ± 135	3797 ± 240	4548 ± 123 cc, s	Muscle and Brain
FSTL-1	2571 ± 71	4090 ± 245 cc	2696 ± 374 ss	Muscle and AT
Apelin	106.9 ± 1.6	92.1 ± 0.9 ccc	89.3 ± 0.8 ccc	Muscle
FABP3	9022 ± 340	12,988 ± 846 cc	6543 ± 663 sss	Muscle, Kidney, Lung and Brain
Osteonectin (SPARC)	4.4 ± 0.2	3.04 ± 0.6 cc	3.07 ± 0.1 cc	Muscle and AT

The results were expressed as means ± SEMs and analyzed using a multifactorial one-way ANOVA followed by Tukey’s test. The number of animals used in each group is *n* = 6. Experimental groups: C, control group; S, selenite group; NS, SeNP group. Significance: vs. C; c *p* < 0.05; cc *p* < 0.01; ccc *p* < 0.001; vs. S; s *p* < 0.05; ss *p* < 0.01; sss *p* < 0.001.

## Data Availability

The data are not publicly available due to the presence of a significant amount of unpublished information. However, they can become accessible to interested scientists upon request.
